# The Prominent Role of the Temporal Lobe in Premenstrual Syndrome and Premenstrual Dysphoric Disorder: Evidence From Multimodal Neuroimaging

**DOI:** 10.3389/fpsyt.2022.954211

**Published:** 2022-06-28

**Authors:** Jingyi Long, Yuejie Wang, Lianzhong Liu, Juan Zhang

**Affiliations:** ^1^Wuhan Mental Health Center, Wuhan, China; ^2^Affiliated Wuhan Mental Health Center, Tongji Medical College of Huazhong University of Science and Technology, Wuhan, China; ^3^Research Center for Psychological and Health Sciences, China University of Geosciences, Wuhan, China

**Keywords:** premenstrual syndrome, premenstrual dysphoric disorder, temporal lobe, magnetic resonance imaging, multimodal neuroimaging

## Abstract

Premenstrual syndrome (PMS) is a group of psychological, physical, and behavioral symptoms that recur with the menstrual cycle, usually occurring a few days before menstruation and ceasing with the onset of menstruation. Premenstrual dysphoric disorder (PMDD) is a severe form of PMS that has been included in a subcategory of depression in the Diagnostic and Statistical Manual of Mental Disorders (DSM-V) according to the latest diagnostic criteria. Patients usually present with mild to moderate emotional and physical symptoms that affect their routine work, social activities, and family lives. The pathogenesis of PMDD remains unclear, and some researchers believe that it is related to fluctuations in ovarian hormone levels. However, the details of the interrelationships and regulating effects between ovarian hormones, symptoms, and the brain need to be more comprehensively determined. Recent studies have revealed some novel findings on PMS and PMDD based on brain morphology, function, and metabolism. Additionally, multiple studies have suggested that PMS and PMDD are closely related to brain structural and functional variations in certain core temporal lobe regions, such as the amygdala and hippocampus. We summarized neuroimaging studies of PMS and PMDD related to the temporal lobe by retrospectively reviewing relevant literature over the past decade. This review contributes to further clarifying the significant role of the temporal lobe in PMS and PMDD and understanding the neurochemical links between hormones, symptoms, and the brain.

## Introduction

Estrogen and progesterone have far-reaching effects on females throughout their lifespan (1). Many female psychiatric disorders are related to reproductive events, such as pubertal depression, premenstrual dysphoric disorder (PMDD), and postpartum depression (2, 3). Approximately 85% of females have experienced mild premenstrual symptoms at least once (4), and 57% of women of childbearing age experienced mild “anger/irritability” or “tearfulness/mood swings” during the premenstrual period (5). About 30–40% of women suffer from premenstrual syndrome (PMS) (6, 7), and 3–8% of females suffer from PMDD (8). PMS incidence is approximately 21.1% in China, and the incidence of PMDD is 2.1% (9). The symptoms range across mental, physical, and behavioral dimensions (10), and difficulty in regulating emotion is the clinical manifestation (4, 6, 11, 12). Remarkably, PMDD is a relatively stable and disabling disease that usually impacts patients' occupational activities, interpersonal relationships, social life, and family life (6, 13) and is responsible for 14.5 million disability-adjusted life years in the United States (8). As a disorder with familial risk, it displays 44 to 56% heritability (14, 15), and the global incidence and associated suicide rate have been continuously increasing (16, 17). In 2013, the American Psychiatric Association classified PMDD as a subcategory of depressive disorders in the Diagnostic and Statistical Manual of Mental Disorders (DSM-V) (4, 18). Currently, diagnosis mainly depends on self-rating scales such as the Daily Record of Severity of Problems. Nevertheless, this diagnostic model is also prone to misdiagnosis of patients (11). Therapeutic modalities include pharmacological, non-pharmacological, and surgical approaches. Usually, selective serotonin reuptake inhibitors are the first-line treatment for PMDD, and ovariectomy is the recommended plan following failure of conservative treatments (6, 10).

In the past three decades, the application of modern science and technology to investigate PMS and PMDD has lagged significantly behind that of other diseases. Today, while neuroimaging research is starting to be applied to these disorders, the neuropathological mechanisms are unclear (11). Even so, mounting evidence has confirmed that certain temporal lobe regions, such as the amygdala and hippocampus, are the most vulnerable regions in PMS and PMDD. This indicates that the temporal lobes have a prominent role in the pathogenesis of PMS and PMDD (19). Furthermore, relevant findings are being continuously updated. Hence, it is indispensable to conduct retrospective analyses and collate results from the most up-to-date literature. Aiming to provide the latest reference for a more comprehensive and in-depth understanding of PMS and PMDD, the present mini-review summarized relevant studies from recent years. These findings are summarized in Table 1.

**Table 1:** Summary of findings of the neuroimaging studies in the menstrual cycle, PMS, or PMDD associated with the temporal lobe.

**Subjects**	**Mode**	**Findings/results**	**References/year**
**Healthy women**	fMRI	Eigenvector centrality in the hippocampus heightened during the LP.	(20) 2020
	fMRI	Irrespective of the task, estradiol boosted hippocampal activation during the pre-ovulatory cycle phase.	(21) 2019
	fMRI	The amygdala and salience network connectivity increased with higher endogenous and synthetic hormone levels.	(22) 2018
	DTI	FA values in the bilateral hippocampus had a significant positive correlation with estrogen.	(23) 2016
	fMRI & sMRI	FC enhanced between the hippocampi and the bilateral superior parietal lobe in the late FP; the bilateral hippocampal volume increased in late FP than in early FP.	(24) 2015
	fMRI	The amygdala response activity increased in the mid-LP; the right anterior hippocampus activity increased in early PF than LP.	(25) 2014
	sMRI	Gray matter volume of the left amygdala increased during the premenstrual phase than in late FP.	(26) 2013
**Women with PMS**	fMRI & sMRI	The volume of the bilateral amygdalae increased; FC increased between the amygdala and the right temporal pole and decreased between the bilateral amygdalae and the right hippocampus.	(27) 2018
	sMRI	PMS patients exhibited increased subcortical volumes of the amygdala.	(28) 2018
	fMRI	The fALFF value of the left hippocampus and left inferior temporal cortex increased at LP.	(29) 2017
	fMRI	FC between the left medial/superior temporal gyri and precentral gyrus within the DMN was enhanced; FC between the middle frontal and parahippocampal gyrus decreased.	(30) 2015
**Women with PMDD**	DTI	FA value increased in the left uncinate fasciculus; volume in the right uncinate fasciculus increased.	(31) 2022
	fMRI	PMDD women had elevated ReHo in the temporal lobe (BA42).	(32) 2021
	fMRI	FC of the anterior temporal lobe decreased across menstrual phases.	(33) 2020
	fMRI	FC of the left middle temporal gyrus and left ECN were significantly enhanced; FC of the left amygdala and cingulate cortex heightened during the FP vs. LP.	(34) 2019
	fMRI & sMRI	FC increased between the left hippocampus and right frontal cortex and decreased between the right hippocampus and right premotor cortex in BDPMDD vs. BD; cortical thickness of the right middle temporal decreased and increased in the left superior temporal gyri BDPMDD vs. BD.	(35) 2018
	fMRI	The amygdala reactivity increased to social stimuli in the LP; altered amygdala reactivity correlated positively with changes in progesterone levels.	(36) 2014
	DTI	Gray matter density significantly increased in the hippocampal cortex and decreased in the parahippocampal gyrus.	(37) 2012
	fMRI	The bilateral amygdala reactivities increased in FP and positively correlated with progesterone serum concentrations; the right amygdala reactivity positively correlated with depression scores in the LP.	(38) 2012

## Article types

Mini-Review.

## Manuscript Formatting

### Critical Regions of the Temporal Lobe in PMS and PMDD

#### Amygdala

It is well-known that the medial temporal lobe (MTL) includes the amygdala, hippocampal region, and entorhinal cortex and is responsible for memory and emotion processing (39). Extensive research has shown that the amygdala exhibits variation in structural and functional tendencies during the menstrual cycle in healthy women (22, 26, 40, 41). In other words, ovarian hormones can trigger the remodeling of the amygdala. For example, structural magnetic resonance imaging (sMRI) analyses demonstrated an increase in gray matter volume of the amygdala from the late luteal phase (LP) to the late follicular phase (FP) in healthy subjects (26). In addition, menstrual cycle-related variations in gray matter volume in the amygdala were associated with the severity of negative affect (19). Furthermore, functional magnetic resonance imaging (fMRI) research showed that progesterone selectively increased amygdala reactivity and regulated the functional coupling of the amygdala with some distant brain regions (22, 40, 41). These alterations may involve regulatory feedback mechanisms related to the menstrual cycle. In contrast, morphological measurements in PMS patients, compared with healthy individuals, indicated significantly enlarged subcortical volumes in the bilateral amygdala (27, 28). Moreover, several studies have verified that enhanced functional activity of the amygdala correlated with emotion dysregulation such as degree of anxiety and depression in LP (12, 38). In terms of functional connectivity (FC), FC between the right amygdala and left anterior cingulate cortex and between the right precentral gyrus and left medial prefrontal cortex exhibited positive correlations with the severity of premenstrual symptoms in PMS females (27). In PMDD women, FC between the left amygdala and cingulate cortex and between the right amygdala and the middle frontal gyrus was markedly strengthened in the FP compared with the LP (34).

The amygdala is located deep in the MTL and plays an essential role in the limbic system and central emotional circuitry (27). Its function involves memory, arousal, alertness, and attention, especially in emotion processing and pain (42–44). Previous findings have shown that anxiety (45), depression (46), and acute and chronic pain (44, 47) could induce abnormalities in the amygdala. Furthermore, the structural and functional alterations of the amygdala in PMS and PMDD are closely associated with emotional regulation (33). Sufficient research evidence suggests that fluctuations in sex hormones, especially progesterone, may be a pivotal factor in brain structural remodeling (2, 38). Given that emotional instability is a core symptom of PMS and PMDD and the amygdala plays a vital role in emotional processing, it is not difficult to explain why neuroimaging abnormalities of the amygdala are so prevalent in patients. The critical question is, how exactly do sex hormones affect the brain? Currently, researchers generally agree that damage to the amygdala leads to aberrations in top-down inhibition processes in the limbic system, which is the core pathological mechanism of PMDD (12, 19, 38, 48). Heightened amygdala responsiveness to social (36) and negative emotional (49) stimuli in PMDD patients confirms the breakdown of another aspect of this inhibitory regulation. Concurrent research on hormones, symptoms, and function surprisingly found that concentrations of sex hormones significantly influence the severity of symptoms and brain activity (36, 38, 48). These findings collectively support the notion that this disruption in top-down inhibition may be the key link in the pathogenesis of PMDD. Although this is the most mainstream academic view on PMDD, there is still some controversy. For instance, Pessoa proposed that the key to emotion regulation is not the limbic system but interactions spanning the entire neuroaxis of a network model of the emotional brain. This network mainly constitutes a functional integration system and emphasizes that abnormal interactions among multiple brain regions lead to PMDD rather than giving the amygdala dominant status in the limbic system (43).

#### Hippocampus and Parahippocampal Gyrus

More recent evidence has shown that the hippocampus is a rich-club hub and the most sensitive region to menstrual cycle changes (50). In healthy females, the hippocampus displayed more intense reactivity during cognitive and affective processes during FP (21, 24), and the responses of the hippocampus were strengthened in the late FP and mid-LP compared with the early FP and late LP during an affective task (25, 51). At the structural level, gray matter volume in the bilateral hippocampi increased in the late FP compared with the early FP (24), and an enlarged volume from LP to FP was positively related to improvements in verbal memory performance (52). Moreover, serum concentrations of estradiol positively correlated with the volume size and fractional anisotropy (FA) values in the hippocampus (23). Overall, it can clearly be seen that the level of estradiol is positively correlated with functional activity (21, 51), volume (24), and white matter integrity (23) of the hippocampus. In contrast, patients with PMDD presented more significantly increases in gray matter density in the hippocampus and decreases in gray matter density in the parahippocampal gyrus than healthy women (37). During the LP, functional activities of the left hippocampus and left inferior temporal cortex were clearly enhanced in PMS (29). Additionally, FC between the right parahippocampal gyrus and middle frontal gyrus in PMS females was weakened (30). It is worth noting that patients with bipolar disorder comorbid with PMDD (BDPMDD) also exhibited diverse abnormalities in FC, such as an enhancement between the left hippocampus and left inferior temporal cortex and a decrease between the right hippocampus and right premotor cortex (35). To date, numerous human and animal studies have confirmed that estrogen has the potential to modify the structure, metabolism, and function of the hippocampus.

How do sex hormones act on the hippocampus? These actions are mainly due to the neurotrophic influence of estradiol (53) and the synaptic remodeling and energy homeostasis effects of progesterone (54). Both estrogen-dependent synaptic remodeling (50) and progesterone-dependent enhancements in synaptic density (54) have been observed in the hippocampus. Indeed, ovarian gonadal hormones can pass through the blood-brain barrier and subsequently affect mood and behavioral patterns by regulating neural activity (2). Notably, sex hormones can profoundly affect the hypothalamus-pituitary-adrenal (HPA) axis and neurotransmitters such as serotonin, dopaminergic, and γ-aminobutyric acid (GABA) (55). These substances are all involved in affective and cognitive processes and in the pathogenesis of depressive disorders (55–57). A rat model of premenstrual depression found that GABA and allopregnanolone expression levels decreased in the hippocampus and that depressive-like symptoms disappeared after ovariectomy and relapsed after hormone priming therapy (58). These findings are in alignment with the theory that neuronal activity in patients with depressive symptoms is being influenced by GABA-mediated inhibition.

Another key question is why the amygdala and hippocampal region are the most susceptible regions in PMS and PMDD. In brief, the amygdala and hippocampus are potential targets of sex hormone effects (50). There is an extensive distribution of estradiol and progesterone receptors in the limbic system, and these receptors are the most densely concentrated in the neurons and glial cells of the amygdala and hippocampus (50, 59). Therefore, ovarian hormones and neurotransmitters and their receptors are crucial factors in inducing structural and functional abnormalities in the amygdala and hippocampal region and subsequently triggering symptoms of PMS and PMDD. As the most vulnerable nodes, the amygdala and hippocampus are expected to be specific neuroimaging targets to assist in diagnosing and treating PMS and PMDD. For instance, it may be that women who do not respond to conservative therapy and refuse hysterectomy and oophorectomy may respond to transcranial magnetic stimulation of these regions, which may alleviate or eradicate symptoms.

#### Other Regions of the Temporal Lobe

In addition to the MTL, other temporal lobe regions are also relevant in PMS and PMDD. Fractional amplitude of low-frequency fluctuation (fALFF) values in the left inferior temporal cortex and left hippocampus were higher in PMS females than in healthy individuals (29). Regional homogeneity (ReHo) in the left inferior temporal cortex was more intense during the LP in PMS females than in healthy women (60). With exposure to emotional stimuli, functional activity in the temporal lobe in PMDD patients was activated to a greater degree than in controls (32). Notably, increased connectivity between the left medial temporal gyrus (MTG) and precentral gyrus negatively correlated with the severity of depression in PMS (30). Furthermore, PMDD patients exhibited hyperconnectivity between the middle temporal gyrus and right amygdala in the FP compared with level of connectivity in the LP (34). Nonetheless, another study observed different manifestations: reduced cortical connectivity across the whole brain, especially within the temporal lobe, and mediation analysis further indicated that 57% of those connections were related to emotional and behavioral symptoms in PMDD (33). The above findings unanimously indicated that the abnormal connectome of the temporal gyrus might mediate deficits in emotion regulation in PMS and PMDD.

Additionally, other evidence suggests that the fusiform gyrus also changes with the menstrual cycle in healthy women. An fMRI study reported abnormal functional activity in the amygdala, fusiform gyrus, inferior temporal gyrus, and middle temporal gyrus (61). Progesterone has been shown to trigger decreases in neural activity in the amygdala and fusiform gyrus, whereas it increases the hippocampal response (62). Moreover, in an emotional recognition task, hypoconnectivity between the fusiform gyrus and amygdala was directly linked to progesterone in healthy subjects (41). However, the existing studies on PMS and PMDD rarely include the fusiform gyrus, and thus, specific neuroimaging characterizations are lacking.

### Temporal Lobe-Related Neural Circuitry in PMS and PMDD

Regions with different properties and locations in the brain form neural circuits through various forms of complex connections. When a particular node is abnormal, other brain regions within the circuit are likely to be disrupted, thereby affecting the overall function of the circuit. In fact, the amygdala and hippocampus occupy core positions in several pivotal neural circuits, such as the hippocampus-amygdala, hippocampus-entorhinal cortex, hippocampus-prefrontal circuit, amygdala-prefrontal cortex, and amygdala-thalamus circuits (63).

Of note, significant variations in several circuits assessed with neuroimaging have also been found in PMS and PMDD. Taking the hippocampus-amygdala circuit as an example, functional activity in this circuit basically coincides with fluctuating levels of ovarian gonadal hormones across menstrual cycle phases in healthy females (61). Namely, the hippocampus-amygdala circuit was positively associated with estradiol levels. As previously described, progesterone and its derivatives regulate emotion and memory processing by modulating the activity of the amygdala, which in turn affects the hippocampus and fusiform gyrus, which are also related to this neural circuit (62). PMS females exhibited weakened FC between the amygdala and right hippocampus and increased FC between the amygdala and right temporal lobe compared with healthy women (27). The above statement is affiliated with the functioning of the hippocampus-amygdala circuit. Particularly, in addition to the amygdala-hippocampus circuit, previous results in PMS and PMDD have focused on the amygdala-prefrontal cortex circuit. Under physiological conditions, the prefrontal lobe maintains bottom-up inhibitory effects on the amygdala and keeps the amygdala in a highly inhibited state, which is vital for maintaining normal emotional behavior. When exposed to estrogen and progestin, inhibitory effects of the prefrontal cortex on the amygdala are weakened, leading to overactivation of the amygdala, which can lead to premenstrual symptoms (12, 19, 38, 48). It is noteworthy that a brain structure study found that the central circuitry of emotion, which includes the amygdala, lateral prefrontal cortex, and orbitofrontal cortex, participated in the pathogenesis of PMS (28). By its nature, abnormal activation of the endocrine system in women and its effects on cognitive and affective circuits may underlie the pathogenesis of PMS and PMDD (33). Admittedly, existing research on neural circuits of PMS and PMDD is insufficient, and a more meticulous analysis of the relevant neural circuits is urgently needed.

### Temporal Lobe-Related Brain Networks in PMS and PMDD

Besides neural circuits, brain regions also constitute neural networks—the execution and maintenance of normal function in bodies rely on homeostasis within and coordination between these networks (64, 65). Previous neuroimaging studies have investigated features of several brain networks in healthy women during the menstrual cycle, but few have focused on PMS and PMDD (33). Earlier findings demonstrated that estrogen and progesterone could mediate sensitivity of mood, cognition, and impairments in brain networks (20). Recently, a refined study gathered MRI data from a woman for 30 consecutive days and repeated it a year later: the recombination and rhythm of the human brain network involved in the menstrual cycle were discovered, and it was confirmed that estradiol plays a crucial role in the brain network (66). Regarding the default mode network (DMN), multiple FC abnormalities, including connections with the parahippocampal gyrus, medial superior temporal gyrus, and MTG, were found in women with PMS (30). In executive control networks (ECN), the connectivity between the left ECN and left middle temporal gyrus was significantly enhanced and closely correlated with emotion regulation in patients with PMDD (34). This finding indicated that the intrinsic network dynamics in the left ECN differ between PMDD patients and healthy subjects. Similarly, a network dynamics study found that fluctuations in ovarian hormone levels during the menstrual cycle affect core brain regions, which may profoundly impact the dynamics of the whole brain network (67). More importantly, a comprehensive topological rearrangement took place in brain networks in healthy women under the influence of sex hormones (31). Topological changes were also present in PMS. Comparing topological parameters in patients from mid-FP to late-LP and healthy subjects demonstrated reduced segregation and enhanced integration among functional brain networks (33). In particular, the specific network for emotional regulation consisting of the amygdala, hippocampus, thalamus, and ventral striatum was closely related to the evaluation and expression of emotion (68). This discovery provided further clues that revealed interactive effects between emotions, brain networks, and sex hormone fluctuations. Moreover, structural research has verified that the fiber tracts responsible for connecting the temporal lobe showed significant microstructural changes, and the FA value in the superior longitudinal fasciculus positively correlated with the severity of premenstrual symptoms in those with PMDD (69). Conversely, unlike other psychiatric disorders, PMDD patients had higher structural integrity of the white matter. This opposite performance could be explained by the neuroprotection of sex hormones positively shaping brain structure in hormone-dependent diseases (69).

**Figure 1:**
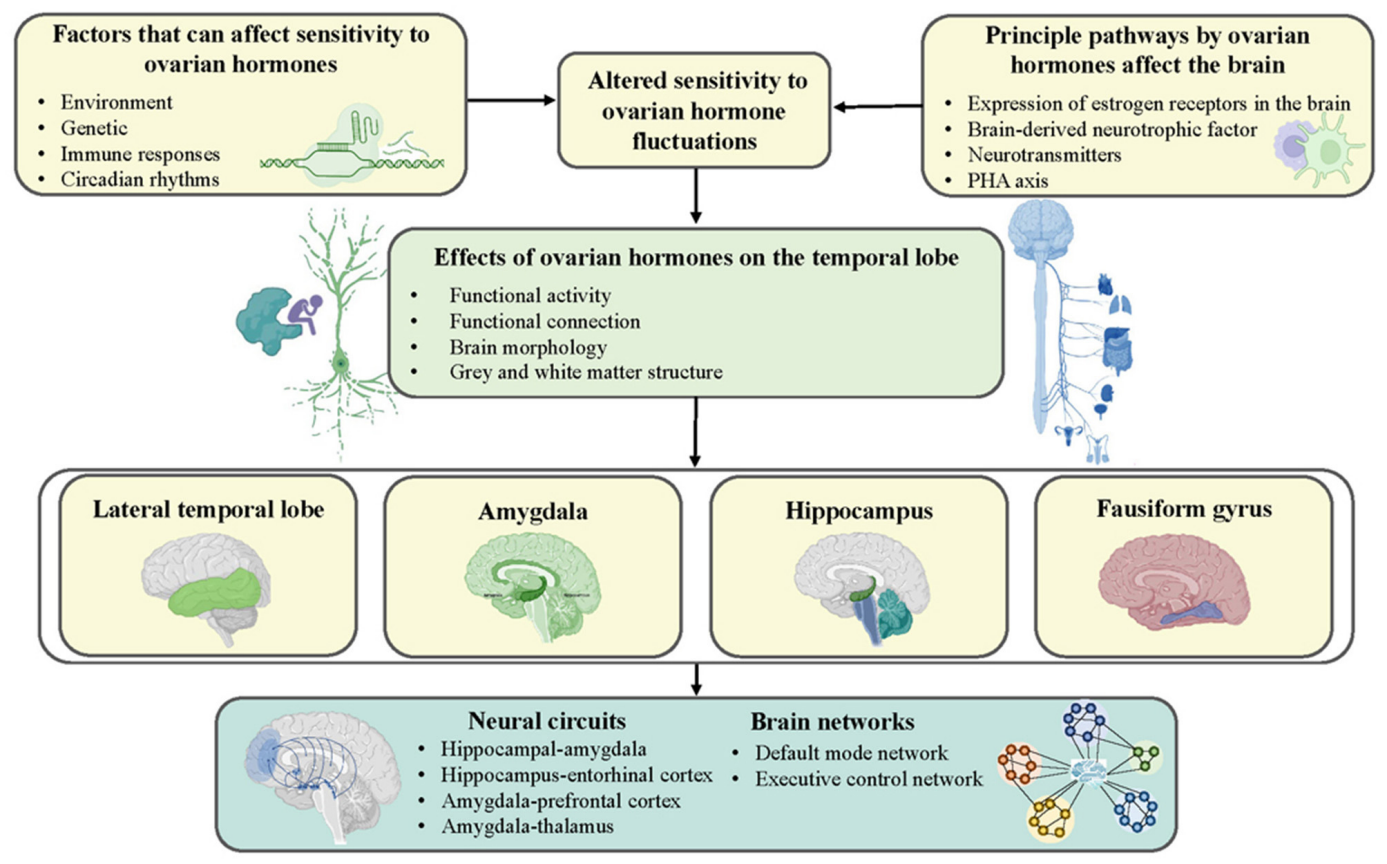
Schematic representation of possible mechanisms by which ovarian hormones contribute to the development of PMS and PMDD. Genetics, environment, immune response, and circadian rhythms increase women's sensitivity to ovarian hormones and shape the temporal lobe by affecting neurotransmitters and hormonal systems.

Overall, insight into the susceptibility and plasticity of network dynamics and topology is necessary to comprehensively explore the neuropathological mechanisms underlying PMS and PMDD (50, 66). However, existing studies of brain network dynamics seriously lack convincing evidence, especially regarding the structural aspects. On the bright side, existing findings evaluating brain networks have mainly converged on temporal lobe-related regions such as the amygdala and hippocampus, which coincided with research on independent brain regions. All these mechanisms are illustrated in Figure 1.

## Conclusion

In general, harmonious covariations between the metabolism, structure, and function of the brain and ovarian gonadal hormone levels during the menstrual cycle exist in healthy females. In contrast, the diverse array of inappropriate manifestations and remodeling of metabolism, structure, and function have occurred in multiple regions in PMS and PMDD women. However, there was a high level of heterogeneity in the current results due to differences in MRI scanning equipment and sequence parameters, analysis methods, and the menstrual phase in which women were scanned. Nevertheless, the existing neuroimaging findings showed high consistency regarding the involvement of temporal lobe regions, such as the amygdala and hippocampus, which confirms the prominent role of the temporal lobe in PMS and PMDD. Furthermore, genetics and environmental factors, abnormal immune responses, and changes in circadian rhythms trigger increased sensitivity to sex hormones, which in turn affect other neurotransmitters and hormonal systems (e.g., brain-derived neurotrophic factor), thereby influencing the temporal lobe and further affecting mental health and behavior (70–72). Therefore, in near future, it is essential to perform research at multiple time points using multimodal neuroimaging techniques, and more attention is warranted on biomarkers, specific neural circuits, structural-functional coupling, vulnerability and controllability of network dynamics, and comorbidity with other psychiatric diseases. Similarly, specific distinctions of menstrual cycle stages and more detailed analyses of brain subregions are needed. If these types of studies can be performed, they will provide a valid foundation of evidence to guide the individualized identification, classification, and treatment of PMS and PMDD.

## Author Contributions

All authors contributed to the article and approved the submitted version.

## Funding

This work was supported by the Health Commission of Wuhan scientific research project (WX19D62 to LL).

## Conflict of Interest

The authors declare that the research was conducted in the absence of any commercial or financial relationships that could be construed as a potential conflict of interest.

## Publisher's Note

All claims expressed in this article are solely those of the authors and do not necessarily represent those of their affiliated organizations, or those of the publisher, the editors and the reviewers. Any product that may be evaluated in this article, or claim that may be made by its manufacturer, is not guaranteed or endorsed by the publisher.
